# Reinvestigation of the crystal structure of lautite, CuAsS

**DOI:** 10.1107/S1600536808004492

**Published:** 2008-02-20

**Authors:** Luca Bindi, Tiziano Catelani, Laura Chelazzi, Paola Bonazzi

**Affiliations:** a Museo di Storia Naturale, Sezione di Mineralogia, Universitá degli Studi di Firenze, Via La Pira 4, I-50121 Firenze, Italy; b Dipartimento di Scienze della Terra, Universitá degli Studi di Firenze, Via La Pira 4, I-50121 Firenze, Italy

## Abstract

The crystal structure of the mineral lautite (copper arsenic sulfide), CuAsS, previously described as either centrosymmetric [*Pnma*; Marumo & Nowacki (1964 ▶). *Schweiz. Miner. Petro. Mitt.* **44**, 439–454] or noncentrosymmetric [*Pna*2_1_; Craig & Stephenson (1965 ▶). *Acta Cryst.* **19**, 543–547], was reinvestigated by means of single-crystal X-ray diffraction. The centrosymmetric structural model reported previously was confirmed, although with improved precision for the atomic coordinates and inter­atomic distances. Lautite shows a sphalerite-derivative structure with a linking of Cu[AsS_3_], As[CuAs_2_S] and S[Cu_3_As] tetra­hedra. All atoms lie on special positions (Wyckoff position 4*c*, site symmetry *m*).

## Related literature

For related literature, see: Craig & Stephenson (1965 ▶); Marumo & Nowacki (1964 ▶); Wyckoff (1963 ▶).

## Experimental

### 

#### Crystal data

AsCuS
*M* *_r_* = 170.54
Orthorhombic, 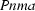

*a* = 11.347 (4) Å
*b* = 3.7533 (7) Å
*c* = 5.453 (1) Å
*V* = 232.24 (10) Å^3^
*Z* = 4
Mo *K*α radiation
μ = 24.00 mm^−1^
*T* = 298 (2) K
0.12 × 0.10 × 0.08 mm

#### Data collection

Bruker *P*4 diffractometer
Absorption correction: ψ scan (North *et al.*, 1968 ▶) *T*_min_ = 0.070, *T*_max_ = 0.150
3824 measured reflections
574 independent reflections
483 reflections with *I* > 2σ(*I*)
*R*_int_ = 0.077
3 standard reflections every 150 reflections intensity decay: none

#### Refinement

*R*[*F* ^2^ > 2σ(*F* ^2^)] = 0.046
*wR*(*F* ^2^) = 0.108
*S* = 1.09
574 reflections
19 parameters
Δρ_max_ = 1.28 e Å^−3^
Δρ_min_ = −1.07 e Å^−3^

### 

Data collection: *XSCANS* (Bruker, 1997 ▶); cell refinement: *XSCANS*; data reduction: *XSCANS*; program(s) used to solve structure: *SHELXS97* (Sheldrick, 2008 ▶); program(s) used to refine structure: *SHELXL97* (Sheldrick, 2008 ▶); molecular graphics: *Xtaldraw* (Downs & Hall-Wallace, 2003 ▶); software used to prepare material for publication: *SHELXL97*.

## Supplementary Material

Crystal structure: contains datablocks global, I. DOI: 10.1107/S1600536808004492/fi2059sup1.cif

Structure factors: contains datablocks I. DOI: 10.1107/S1600536808004492/fi2059Isup2.hkl

Additional supplementary materials:  crystallographic information; 3D view; checkCIF report

## Figures and Tables

**Fig. 1.**
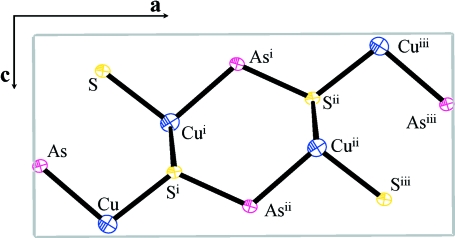
The crystal structure of lautite down [010]. Displacement parameters are drawn at the 70% probability level. The unit-cell is outlined. Symmetry codes are: (i) -x + 1/2; -y; z + 1/2; (ii) x + 1/2; -y + 1/2; -z + 1/2; (iii) -x; y + 1/2; -z.

**Fig. 2.**
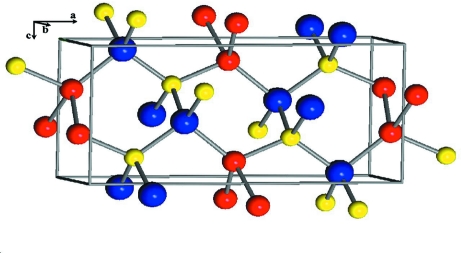
The crystal structure of lautite showing the linking of Cu[AsS3], As[CuAs2S] and S[Cu3As] tetrahedra. Blu, red and yellow circles indicate Cu, As and S atoms, respectively. The unit-cell is outlined.

## supplementary crystallographic information

### Comment

The crystal structure of lautite was solved by Marumo & Nowacki (1964) in the space group *Pnma* (*R* = 9.0%) and by Craig & Stephenson (1965) in the space group *Pna*2_1_ (*R* = 13.7%) by means of photographic data and three-dimensional Patterson-function. The low quality of the structural data given by these authors, however, did not allow to obtain an anisotropic model of the structure. Nevertheless, the topologies and interatomic distances of both centrosymmetric and non-centrosymmetric models are very similar.

Although the structural results obtained by Craig & Stephenson (1965) indicate the acentricity of the structure of CuAsS, no clear crystal-chemical reason for the choice of a noncentrosymmetric arrangement was given. To help resolve the concerns relating to the structure of natural lautite, we present new crystal structure data for lautite from its type locality (*i.e.*, Marienberg, Saxony, Germany).

The centrosymmetric structural model previously reported by Marumo & Nowacki (1964) was confirmed, although a higher precision of refinement was achieved (e.s.d. improved by a factor of two) and refinement with anisotropic displacement parameters could be performed (Fig. 1). All atoms lie on special positions (Wyckoff position 4c, site symmetry m). Lautite shows a sphalerite-derivative structure with a linking of Cu[AsS_3_], As[CuAs_2_S] and S[Cu_3_As] tetrahedra (Fig. 2). Within the framework, the As atoms form zigzag As—As chains along [010] exhibiting As—As bond distances [2.4965 (8) Å] and angles [97.48 (4)°] resembling the covalent As—As linkage observed within the sheets of the crystal structure of arsenic (Wyckoff, 1963).

### Experimental

A crystal was selected from a natural specimen belonging to the Mineralogical Collection of the Natural History Museum of Florence (catalogue number 44202/G).

### Refinement

The crystal structure refinement was performed starting from the atomic coordinates reported by Marumo & Nowacki (1964). Convergence was rapidly obtained for an anisotropic model of the structure.

### Crystal data

**Table:**

AsCuS	*F*_000_ = 312
*M**_r_* = 170.54	*D*_x_ = 4.878 Mg m^−^^3^
Orthorhombic, *P**n**m**a*	Mo *K*α radiation λ = 0.71073 Å
Hall symbol: -P 2ac 2n	Cell parameters from 38 reflections
*a* = 11.347 (4) Å	θ = 12.5–24.3º
*b* = 3.7533 (7) Å	µ = 24.00 mm^−^^1^
*c* = 5.453 (1) Å	*T* = 298 (2) K
*V* = 232.24 (10) Å^3^	Block, black
*Z* = 4	0.12 × 0.10 × 0.08 mm

### Data collection

**Table:**

Bruker P4 diffractometer	*R*_int_ = 0.077
Radiation source: fine-focus sealed tube	θ_max_ = 35.0º
Monochromator: graphite	θ_min_ = 3.6º
*T* = 298(2) K	*h* = −18→18
ω scans	*k* = −6→6
Absorption correction: ψ scan(North *et al.*, 1968)	*l* = −8→8
*T*_min_ = 0.070, *T*_max_ = 0.150	3 standard reflections
3824 measured reflections	every 150 reflections
574 independent reflections	intensity decay: none
483 reflections with *I* > 2σ(*I*)	

### Refinement

**Table:**

Refinement on *F*^2^	Primary atom site location: structure-invariant direct methods
Least-squares matrix: full	Secondary atom site location: difference Fourier map
*R*[*F*^2^ > 2σ(*F*^2^)] = 0.046	*w* = 1/[σ^2^(*F*_o_^2^) + (0.0856*P*)^2^ + 1.9844*P*] where *P* = (*F*_o_^2^ + 2*F*_c_^2^)/3
*wR*(*F*^2^) = 0.108	(Δ/σ)_max_ < 0.001
*S* = 1.09	Δρ_max_ = 1.28at 0.0736 0.2500 0.3457 (0.68 Å from As) e Å^−^^3^
574 reflections	Δρ_min_ = −1.07at 0.0052 0.0883 0.4402 (0.78 Å from As) e Å^−^^3^
19 parameters	Extinction correction: none

### Special details

**Table:**

Geometry. All e.s.d.'s (except the e.s.d. in the dihedral angle between two l.s. planes) are estimated using the full covariance matrix. The cell e.s.d.'s are taken into account individually in the estimation of e.s.d.'s in distances, angles and torsion angles; correlations between e.s.d.'s in cell parameters are only used when they are defined by crystal symmetry. An approximate (isotropic) treatment of cell e.s.d.'s is used for estimating e.s.d.'s involving l.s. planes.
Refinement. Refinement of *F*^2^ against ALL reflections. The weighted *R*-factor *wR* and goodness of fit *S* are based on *F*^2^, conventional *R*-factors *R* are based on *F*, with *F* set to zero for negative *F*^2^. The threshold expression of *F*^2^ > σ(*F*^2^) is used only for calculating *R*-factors(gt) *etc*. and is not relevant to the choice of reflections for refinement. *R*-factors based on *F*^2^ are statistically about twice as large as those based on *F*, and *R*- factors based on ALL data will be even larger.

### Fractional atomic coordinates and isotropic or equivalent isotropic displacement parameters (Å^2^)

**Table:**

	*x*	*y*	*z*	*U*_iso_*/*U*_eq_	
Cu	0.17454 (7)	0.2500	0.06264 (18)	0.0165 (2)	
As	0.01373 (5)	0.2500	0.35177 (11)	0.00894 (18)	
S	0.16576 (12)	0.7500	0.8196 (3)	0.0100 (3)	

### Atomic displacement parameters (Å^2^)

**Table:**

	*U*^11^	*U*^22^	*U*^33^	*U*^12^	*U*^13^	*U*^23^
Cu	0.0171 (3)	0.0168 (4)	0.0157 (4)	0.000	−0.0015 (3)	0.000
As	0.0105 (3)	0.0084 (3)	0.0080 (3)	0.000	0.00083 (17)	0.000
S	0.0108 (5)	0.0114 (5)	0.0079 (5)	0.000	−0.0005 (4)	0.000

### Geometric parameters (Å, °)

**Table:**

Cu—S^i^	2.2908 (16)	As—As^v^	2.4965 (8)
Cu—S^ii^	2.2996 (10)	S—As^iv^	2.2408 (16)
Cu—S^iii^	2.2996 (10)	S—Cu^vi^	2.2908 (16)
Cu—As	2.4114 (11)	S—Cu^vii^	2.2996 (10)
As—S^iv^	2.2408 (16)	S—Cu^viii^	2.2996 (10)
As—As^iv^	2.4965 (8)		
			
S^i^—Cu—S^ii^	112.76 (3)	S^iv^—As—As^v^	99.01 (4)
S^i^—Cu—S^iii^	112.76 (3)	Cu—As—As^v^	121.19 (3)
S^ii^—Cu—S^iii^	109.39 (7)	As^iv^—As—As^v^	97.48 (4)
S^i^—Cu—As	101.46 (5)	As^iv^—S—Cu^vi^	117.64 (7)
S^ii^—Cu—As	110.12 (4)	As^iv^—S—Cu^vii^	106.24 (5)
S^iii^—Cu—As	110.12 (4)	Cu^vi^—S—Cu^vii^	108.56 (4)
S^iv^—As—Cu	114.52 (5)	As^iv^—S—Cu^viii^	106.24 (5)
S^iv^—As—As^iv^	99.01 (4)	Cu^vi^—S—Cu^viii^	108.56 (4)
Cu—As—As^iv^	121.19 (3)	Cu^vii^—S—Cu^viii^	109.39 (7)

Symmetry codes: (i) −*x*+1/2, −*y*+1, *z*−1/2; (ii) *x*, *y*−1, *z*−1; (iii) *x*, *y*, *z*−1; (iv) −*x*, −*y*+1, −*z*+1; (v) −*x*, −*y*, −*z*+1; (vi) −*x*+1/2, −*y*+1, *z*+1/2; (vii) *x*, *y*+1, *z*+1; (viii) *x*, *y*, *z*+1.
